# Preliminary development of a measure of parental behavioral responses to everyday pains in young children: the PREP

**DOI:** 10.1097/PR9.0000000000001154

**Published:** 2024-04-03

**Authors:** Perri R. Tutelman, Christine T. Chambers, Jennifer A. Parker, Samantha J. Eisen, Melanie Noel

**Affiliations:** a Department of Psychology & Neuroscience, Dalhousie University, Halifax, NS, Canada; b Centre for Pediatric Pain Research, IWK Health, Halifax, NS, Canada; Departments of c Oncology and; d Psychology, University of Calgary, Calgary, AB, Canada; e Department of Pediatrics, Dalhousie University, Halifax, NS, Canada; f Children's Hospital Research Institute, Calgary, AB, Canada; g Hotchkiss Brain Institute, University of Calgary, Calgary, AB, Canada; h Owerko Centre, Calgary, AB, Canada

**Keywords:** Everyday pains, Children, Toddler, Preschooler, Parent, Questionnaire, Distress, Acute pain

## Abstract

**Introduction:**

Everyday pains are experienced frequently by young children. Parent responses shape how young children learn about and experience pain. However, research on everyday pains in toddlers and preschoolers is scarce, and no self-report measures of parent responses to their child's pain exist for this age group.

**Objectives:**

The objective of this study was to develop a preliminary self-report measure of parent behavioral responses to everyday pains in the toddler and preschool years (the PREP) and examine its relationship with child age, sex, and parent and child distress.

**Methods:**

Items for the PREP were based on a behavioural checklist used in a past observational study of caregiver responses to toddler's everyday pains. Parents (N = 290; 93% mothers) of healthy children (47.9% boys) between 18 and 60 months (M_age_ = 34.98 months, SD = 11.88 months) completed an online survey of 46 initial PREP items, demographic characteristics, and their child's typical distress following everyday pains. An exploratory factor analysis was performed on the PREP items that describe observable actions parents may take in response to their young child's everyday pains.

**Results:**

The final solution included 10 items across 3 factors: *Distract*, *Physical Soothe*, and *Extra Attention* and explained 60% of the model variance. All PREP subscales were related to child distress; only *Physical Soothe* and *Extra Attention* were related to parent distress.

**Conclusion:**

This study was a preliminary step in the development and testing of a new self-report measure of parental responses to everyday pains during early childhood.

## Abstract 2

Supplemental Digital Content is Available in the Text.

The PREP is a relatively short (10 item) measure assessing self-reported observable parent responses to their child's everyday pains.

## 1. Introduction

Everyday pains (EDPs; ie, minor bumps and scrapes incurred during everyday activities) are experienced frequently, particularly early in development when children begin walking and attending school.^13,23,32^ Everyday pains provide young children with opportunities for learning about pain during critical developmental periods. This learning could be influenced by parents' responses to painful events. Nevertheless, research examining EDPs is scarce, and no self-report measures of parent responses to young children's EDPs exist.

Among infant, child, and adolescent samples, parent behaviors that direct attention toward pain (eg, verbal reassurance) or that reinforce pain behaviors (eg, protectiveness) are linked to increased child pain, distress, and disability^2–4,7,9,22,24,32^ and have been conceptualized within an operant learning framework.^6,14,19^ However, it cannot be assumed that parent responses have the same effect in clinical and naturalistic settings^25,33^ and in children with and without chronic conditions. To date, only a handful of studies have examined EDPs in children aged 1 to 7 years.^13,15,23,26,27,32^ Findings reveal that EDPs are frequent, sometimes evoke distress and caregiver responses, and are influenced by child age and sex.^25^ In most studies, observers used observational checklists (eg, the Dalhousie Everyday Pain Scale) to code caregivers' responses to young children's EDPs.^13,15,23,26,32^ More recently, parents have used electronic diaries to self-report their child's pain and their own distress. However, this field is limited by the lack of self-report measures of caregiver *responses* to EDPs in young children's.

Informed by parent behaviors observed in a study of parent responses to young children's EDPs,^23^ we developed a preliminary self-report measure of parent behavioral responses to EDPs during the toddler and preschool years (the PREP) and examined its relationship with child age, sex, and parent and child distress.

## 2. Methods

### 2.1. Participants

Parents (N = 294) participated in an online survey which took approximately 30 minutes to complete. Participants missing >3 items were excluded, leaving a final sample of N *=* 290 parents, which is consistent with best practice recommendations for exploratory factor analyses (EFAs).^5,18^ Participants were primarily mothers (93%) of children aged 18 to 60 months (Table 1). Inclusion criteria were (1) have >1 child between 18–60 months, (2) able to read and understand English, and (3) have Internet access. The Research Ethics Board approved this study.

**Table 1:** Participant characteristics.

Variable	Parent report (N = 290)
Child age (mo), mean (SD), (N *=* 285)	34.98 (11.88)
Child sex, N (%)	
Male	139 (47.9)
Female	148 (51.0)
No response	3 (1.0)
Child race, N (%)	
White	263 (90.7)
Asian	3 (1.0)
Arab	2 (0.7)
Black	5 (1.7)
Latin American	1 (0.3)
Native/Aboriginal	1 (0.3)
Other	14 (4.8)
No response	1 (0.3)
Parent age (y), mean (SD), (N = 275)	33.60 (4.64)
Parent relationship to child, N (%)	
Mother	270 (93.1)
Father	16 (5.5)
Stepmother	2 (0.7)
No response	2 (0.7)
Parent race, N (%)	
White	271 (93.4)
Asian	1 (0.3)
Arab	2 (0.7)
Black	3 (1.0)
Latin American	1 (0.3)
Native/Aboriginal	1 (0.3)
Other	7 (2.4)
No response	4 (1.4)
Parent marital status, N (%)	
Married/common law	259 (89.3)
Divorced/separated	12 (4.1)
Never married	15 (5.2)
Remarried	2 (0.7)
No response	2 (0.7)
Parent highest level of education, N (%)	
Some high school	2 (0.7)
High school graduate	21 (7.2)
Trade school/community college	59 (20.3)
University graduate	100 (34.5)
Grad/professional school	107 (26.9)
No response	1 (0.3)
Parent and child everyday pain distress, mean (SD)*	
Parent distress (N *=* 289)	1.21 (1.04)
Child distress (N *=* 287)	2.58 (1.28)

* Possible scores ranged from 0 to 5 with higher scores indicating more distress or pain.

### 2.2. Measures

#### 2.2.1. Demographics

Parents reported their age, racialized identity/ethnicity, relationship to child, marital status, and educational level. They reported on their child's age, sex, and racialized identity/ethnicity.

#### 2.2.2. Everyday pains in young children

The Parent Responses to Everyday Pains (the PREP) questionnaire assessed parents' self-reported responses to their child's EDP experiences. Everyday pain experiences were defined as instances when a child's body comes into contact with another person or object and results in either: (1) a distress (eg, crying), anger, or protective (eg, help-seeking) reaction from the child or (2) the parent judges that the child experienced at least momentary, minor discomfort. Items were based on the Dalhousie Everyday Pains Scale-Revised, a behavioural checklist used in an observational study of parent responses to toddler's EDPs.^13^ Using a 5-point Likert scale ranging from 1 (“never”) to 5 (“usually”), parents were asked to indicate how often they engage in 46 responses. Possible responses were separated into 28 verbal statements (eg, “Tell him/her to stop crying”) and 18 observable actions (eg, “Distract child with a toy”) (Supplementary Material, http://links.lww.com/PR9/A229). Given that the empirical study from which this measure was based^23^ coded only parents' *observable* actions and not verbal statements (due to the busy nature of the play setting), only items reflecting observable responses to children's pain were included in the analyses.

#### 2.2.3. Parent and child distress

Parents were asked to indicate how distressed they and their child are, on average, following the child's EDP experiences on a 6-point Likert scale ranging from 0 (“low distress”) to 5 (“high distress”).

### 2.3. Procedure

Parents were recruited to participate in the study through online and newspaper advertisements, posters, and email invitations through hospitals, community centers, and social media. Parents provided consent to participate and were entered into a draw for 1 of 3 $100 gift certificates.

### 2.4. Data analysis

Analyses were conducted using SPSS (v22). An EFA was performed on the 18 observable PREP items. Similar to previous pediatric pain studies,^12,24^ iterated principal axis factoring using a nonorthogonal (oblique) rotation method was used as this method allows factors to be correlated, resulting in a more accurate, reproducible solution.^11^ Eigenvalues ≥1,^10^ >50% of variance explained,^17,29,31^ and the initial scree plot were used to select a factor structure.

To be retained in the model, individual items were required to have a primary loading above the recommended threshold of 0.32^31^ and items that cross-loaded on the same factor had to have loadings of at least twice the value of the other.^12^ We systematically removed items one at a time and reran analyses each time, applying these criteria at each stage. Models were run until a clean solution was obtained. Final clean solutions were examined and compared. The superiority of models was judged based on interpretability and overall variance accounted for. Factor solutions were required to have at least 3 items loading onto each subscale.^10^

Bivariate correlations were conducted between the final subscales and child age, sex, and child and parent distress.

## 3. Results

The initial EFA resulted in 5 factors with initial eigenvalues above 1.0% and 63.4% variance explained. The scree plot suggested a 3-factor solution; therefore, 5-factor, 4-factor, and 3-factor models were tested by forcing each respective solution. In the initial EFA, the 4-factor and 3-factor models accounted for 57.5% and 50.4% of the variance, respectively.

After systematically removing poorly loading and highly cross-loading items for each model, final clean solutions were obtained. The 5-factor solution accounted for 55.6% of the total model variance; however, 2 of the factors included only 2 items. The 4-factor model explained 56.6% of the total model variance; however, one of the subscales was indicative of an *absence* of soothing behaviors, which was deemed not as interpretable as the 3-factor model. The 3-factor model was deemed to be superior to the 4-factor and 5-factor models based on both interpretability and total variance explained. The final solution included 10 items across 3 factors: *Distract* (4 items), *Physical Soothe* (3 items), and *Extra Attention* (3 items). The final 3-factor solution explained 60% of the model variance with each factor explaining 33.2%, 19.6%, and 7.2% of the variance, respectively. The final model had primary loadings of >0.50 (Table 2).

**Table 2:** Exploratory factor analysis factor loading matrix for parent behavioral responses to everyday pains in the toddler and preschool years items for the final 3-factor model.

PREP item	Distract	Physical soothe	Extra attention
1. Give him/her hugs and/or kisses		0.61	
2. Pick him/her up		0.76	
3. Cuddle him/her		0.97	
4. Distract him/her with a toy	0.81		
5. Point to something else to distract him/her	0.95		
6. Ask him/her to help you with a new activity	0.71		
7. Take him/her out of the play setting			0.51
8. Give him/her extra attention			0.68
9. Do something special to make him/her feel better			0.67
10. Try to engage him/her in a new play activity	0.62		

The following items were removed: show no reaction/try to show no reaction; leave the room; pay no attention until he/she goes back to playing; rub the hurt area better; kiss the “boo-boo” or hurt area better; stay closer to him/her; keep close watch on his/her activities so that he/she does not get hurt again; gently tap or swat his/her bottom.

PREP, parent behavioral responses to everyday pains in the toddler and preschool years.

Internal consistency was high for the *Distract* (0.87) and *Physical Soothe* (0.83) subscales and acceptable for the *Extra Attention* subscale (0.69). Table 3 presents mean subscale and item scores.

**Table 3:** Mean (SD) parent behavioral responses to everyday pains in the toddler and preschool years scores by subscale (N = 290).

Parental response	Mean (SD)	Mean (SD)
Physical soothe		3.87 (0.79)
Hug/kiss child	4.23 (0.82)	
Pick up child*	3.57 (0.98)	
Cuddle child	3.81 (0.96)	
Distract		2.90 (0.82)
Distract child with a toy	2.87 (0.95)	
Point to something else to distract child*	2.92 (1.02)	
Ask child to help with a new activity	2.92 (0.97)	
Try to engage child in a new activity	2.98 (0.94)	
Extra attention		2.45 (0.73)
Take child out of play setting*	2.04 (8.6)	
Give child extra attention	2.83 (1.03)	
Do something special to make the child feel better	2.47 (0.92)	

Possible scores ranged from 1 to 5; higher scores indicate more frequent engagement in pain response.

* n = 289 responses.

Bivariate correlations revealed that higher scores on the *Distract* subscale were positively associated with child (but not parent) distress during EDP events (*r* = 0.12, *P* < 0.05). Higher scores on the *Physical Soothe* and *Extra Attention* subscales were positively associated with child (*r* = 0.21, *P* < 0.001; *r* = 0.21, *P* < 0.001) and parent distress (*r* = 0.21, *P* < 0.001; *r* = 0.32, *P* < 0.001). *Physical Soothe* scores were negatively related to child age (*r* = −0.12, *P* < 0.05). No subscale scores differed significantly by child sex (*P* > 0.05).

## 4. Discussion

This study describes the development and preliminary testing of a 10-item self-report measure of parental responses to EDPs during the toddler and preschool years. While some items (*Distract, Extra Attention*) are consistent with responses assessed on measures for use with older youth,^24^ others (*Physical Soothe*) are more characteristic of earlier developmental periods. Parents of younger children in our previous study were more likely to engage in more physical soothing in response to their children's EDPs.^23^ All PREP subscales were related to child distress in the context of EDPs; however, relationships were small and require further investigation. While higher *Physical Soothe* and *Extra Attention* scores were related to greater parent distress to children's EDPs, distracting responses were not. These findings may be conceptualized within Goubert's pain empathy model,^16^ which posits that parents who experience self-oriented distress on observing their child in pain may be less able to engage in responses to child pain that can foster adaptive coping. Distracting responses have been linked to less child pain and distress in laboratory-based and clinical pain settings among infants to older adolescents.^1,8,21,28^ Physical soothing and providing extra attention may be driven by heightened parental distress and a self-oriented response; however, further research is needed.

This study has limitations. The PREP includes only observable parent responses to children's EDPs in a sample of primarily mothers. Parents' verbal behaviors (eg, reassurance) have been linked to child pain outcomes in other acute pain settings^7,22^; further measure development work examining verbal responses is warranted. The utility of the PREP as a nonverbal behavioral measure of parent responses might be limited to younger developmental periods. Effect sizes of the relationships between the PREP subscales and child and parent distress were small; therefore, practical implications cannot be definitively drawn. Research is needed with larger samples to further validate the PREP using confirmatory factor analysis and relationships to other measures of parent responses to child pain. Future research using the PREP with ecological momentary assessment methods could be used to assess variation in parental distress and responses to EDPs across contexts.

## Disclosures

The authors declare that the research was conducted in the absence of any commercial or financial relationships that could be constructed as a potential conflict of interest.

## Supplementary Material

SUPPLEMENTARY MATERIAL

## Supplemental digital content

Supplemental digital content associated with this article can be found online at http://links.lww.com/PR9/A229.
