# Supplementary material for: Preliminary development of a measure of parental behavioral responses to everyday pains in young children: the PREP
Source: Pain Rep. 2024 Apr 3;9(3):e1154. doi: 10.1097/PR9.0000000000001154 (PMC10994502; doi:10.1097/PR9.0000000000001154)
Supplement: SUPPLEMENTARY MATERIAL [file painreports-9-e1154-s001.pdf]

Parental Behavioral Responses to Everyday Pains in Young Children (the PREP)

Everyday pains are the minor bumps and scrapes that children experience as part of their daily activities and include things such as falling down, bumping into things, and tripping.

So, an *everyday pain experience* is when a child's body comes into contact with another person or object and results in either a) a distress (e.g., crying), anger, or protective (e.g., seeking help from a parent) reaction from the child or b) the parent judges that the child experienced at least momentary, minor discomfort.

Please indicate how often you do each of the following activities after your child experiences everyday pains, in general:

	Never	Seldom	Sometimes	Often	Usually
1. Give him/her hugs and/or kisses	0	1	2	3	4
2. Pick him/her up	0	1	2	3	4
3. Cuddle him/her	0	1	2	3	4
4. Distract him/her with a toy	0	1	2	3	4
5. Point to something else to distract him/her	0	1	2	3	4
6. Ask him/her to help you with a new activity	0	1	2	3	4
7. Take him/her out of the play setting	0	1	2	3	4
8. Give him/her extra attention	0	1	2	3	4
9. Do something special to make him/her feel better	0	1	2	3	4
10. Try to engage him/her in a new play activity	0	1	2	3	4

Supplemental Material.

Preliminary Development of a Measure of Parental Behavioral Responses to Everyday Pains in Young Children: The PREP

Tutelman et al.

Initial Verbal Items (N = 28)

1. Nothing
2. Provide reassurance (e.g., "You'll be okay" or "it's okay")
3. Apologize/express regret (e.g., "I'm sorry that you got hurt")
4. Sympathize/empathize (e.g., "I know how much that hurts")
5. Encourage him/her to do something else
6. Encourage him/her to try to think about something else
7. Talk to him/her about something unrelated to the hurt
8. Encourage him/her to do something s/he likes
9. Ask him/her to help you with a new activity
10. Talk to him/her about the hurt or pain
11. Tell him/her to stop crying
12. Tell him/her to be more careful/not to be careless
13. Check in with him/her (e.g., "are you okay?")
14. Tell him/her that big boys/girls don't cry
15. Remind him/her that crying won't make it better
16. Tell him/her, "You're okay, go back to playing"
17. Tell him/her to stop crying/complaining or you will take away privileges (toys or play time)
18. Tell him/her, "that's what happens when you don't pay attention"
19. Tell jokes or tickle him/her
20. Praise him/her
21. Shush him/her (e.g., "shh, shh")
22. Ask him/her if it hurt
23. Say, "I told you not to do that"
24. Offer to read him/her a story
25. Encourage him/her to breathe deeply
26. Get upset with him/her
27. Give him/her a time- out/send to room to calm down
28. Offer unnecessary medical attention just to make him/her feel better (e.g., band-aid)

Initial Non-Verbal Items (N = 18)

1. Show no reaction/try to show no reaction
2. Leave the room
3. Pay no attention until he/she goes back to playing
4. Rub the hurt area better
5. Give him/her hugs and/or kisses
6. Kiss the 'boo-boo' or hurt area better
7. Stay closer to him/her

Supplemental Material.

8. Pick him/her up
9. Cuddle him/her
10. Distract him/her with a toy
11. Point to something else to distract him/her
12. Ask him/her to help you with a new activity
13. Take him/her out of the play setting
14. Give him/her extra attention
15. Do something special to make him/her feel better
16. Keep a close watch on his/her activities so that he/she doesn't get hurt again
17. Try to engage him/her in a new play activity
18. Gently tap or swat his/her bottom
